# Experimental Evaluation of Perfluorocarbon Aerosol Generation with Two Novel Nebulizer Prototypes

**DOI:** 10.3390/pharmaceutics11010019

**Published:** 2019-01-05

**Authors:** Iñigo Aramendia, Unai Fernandez-Gamiz, Alberto Lopez-Arraiza, Carmen Rey-Santano, Victoria Mielgo, Francisco Jose Basterretxea, Javier Sancho, Miguel Angel Gomez-Solaetxe

**Affiliations:** 1 Nuclear Engineering and Fluid Mechanics Department, University of the Basque Country UPV/EHU, 01006 Vitoria-Gasteiz, Araba, Spain; inigo.aramendia@ehu.eus (I.A.); javier.sancho@ehu.eus (J.S.); 2 Department of Nautical Science and Marine Systems, University of the Basque Country UPV/EHU, 48013 Portugalete, Bizkaia, Spain; alberto.lopeza@ehu.eus (A.L.-A.); miguel.solaetxe@ehu.eus (M.A.G.-S.); 3 Animal Research Unit, BioCruces Health Research Institute, 48903 Barakaldo, Bizkaia, Spain; mariacarmen.reysantano@osakidetza.eus (C.R.-S.); victoriaeugenia.mielgoturuelo@osakidetza.eus (V.M.); 4 Department of Physical Chemistry, University of the Basque Country UPV/EHU, 48940 Leioa, Bizkaia, Spain; franciscojose.basterretxea@ehu.eus

**Keywords:** drug delivery, nebulizer, aerosol, aerodynamic particle sizer, particle size distribution

## Abstract

The potential of non-invasive ventilation procedures and new minimally invasive techniques has resulted in the research of alternative approaches as the aerosolization for the treatment of respiratory distress syndrome (RDS). The aim of this work was to design two nebulizer prototypes and to evaluate them studying the particle size distribution of the inhaled droplets generated with distilled water and two perfluorocarbons (PFCs). Different experiments were performed with driving pressures of 1–3 bar for each compound. An Aerodynamic Particle Sizer was used to measure the aerodynamic diameter (Da), the mass median aerodynamic diameter (MMAD) and the geometric standard deviation (GSD). The results showed that both prototypes produced heterodisperse aerosols with Da mean values in all cases below 5 µm. The initial experiments with distilled water showed MMAD values lower than 9 µm and up to 15 µm with prototype 1 and prototype 2, respectively. Regarding the PFCs, relatively uniform MMAD values close to 12 µm were achieved. The air delivery with outer lumens of prototype 1 presented more suitable mass distribution for the generation and delivery of a uniform aerosol than the two half-circular ring geometry proposed in the prototype 2.

## 1. Introduction

Respiratory distress syndrome (RDS), which results from a lack of pulmonary surfactant, remains as the main cause of mortality and morbidity in preterm infants [1]. The major components of surfactant are lipids which are responsible for adjusting the surface tension during the breathing cycle, as described by Nkadi et al. [2]. Parra et al. [3] made a detailed review of the composition, structure and properties of the surfactant and problems due to its inactivation. The current clinical surfactant replacement therapy involves endotracheal intubation and the application of mechanical ventilation, which are techniques that may lead to lung injury (Jobe et al. [4]). Therefore, clinicians are constantly looking for new soft approaches in order to minimize the invasive intervention (Herting et al. [5]). Non-invasive ventilation procedures, such as continuous positive airway pressure (CPAP), along with new minimally invasive surfactant therapy (MIST) techniques have emerged as alternatives for the treatment of RDS [6,7]. They can be classified into four main groups: pharyngeal surfactant instillation, administration via a laryngeal mask, surfactant instillation via a thin catheter and surfactant aerosolization [8]. More et al. [9] carried out a meta-narrative review of the studies using these types of techniques comparing the safety and feasibility between them.

Verder et al. [10] introduced in 1994 the INSURE (INtubation SURfactant Extubation) technique, which consists of intubating the preterm infant for surfactant administration with quick extubation to nCPAP (nasal Continuous Positive Airways Pressure). Dani et al. [11] confirmed that INSURE procedure is a safe and feasible method to be applied in preterm infants with a high percentage of success. Recently, Aguar et al. [12] reported that MIST was as effective as INSURE in avoiding the need for further mechanical ventilation.

According to Dolovich et al. [13], aerosol drug delivery devices can be classified in three different types: pressurized metered-dose inhalers (pMDIs), dry powder inhalers (DPI) and nebulizers. Rubin et al. [14] described the aerosol therapy with each one of these devices and the major mechanisms of aerosol deposition. Nebulizers convert a liquid in solution or suspension into small droplets. They are widely used for hospital emergency care and pediatric patients, as they do not require specialized inhalation maneuvers apart from the patient’s spontaneous breathing. Therefore, in contrast to pMDIs or DPIs, they can be used for patients who are unable to control their breathing or consciously follow instructions, as preterm infants affected with RDS [15]. O’Callaghan and Barry [16] analyzed the nebulizers operation principle and described some of the most important parameters to define an aerosol as well as some methods to measure particle size, aerosol deposition and nebulizer output. Nebulizers are classified by the type of energy used for the disintegration of the liquid. Pressure nebulizers convert liquid pressure into kinetic energy that causes the liquid to break into droplets. There are two types of pressure nebulizers, jet nebulizers and swirl nebulizers. Jet nebulizers are based on the Bernoulli principle, where a pressurized flow of gas, generally air, is directed through a constricted orifice where the velocity of the airflow is increased to create a jet stream. This jet stream creates a sub-atmospheric pressure zone (vacuum) which draws the fluid up the capillary tube. The impact of a jet stream with the liquid and the extreme difference in velocity between them produces the aerosol particles or droplets [15,17]. Swirl nebulizers cause the liquid to spin as it exits the nozzle, forming a hollow cone that facilitates the breakup of the liquid. Another type is the pneumatic nebulizer, which uses the energy from compressed air to break up a liquid stream. Ultrasonic nebulizers remove the need for a compressed air source and, instead, a high-frequency vibration generated by a piezoelectric crystal is used to the formation of droplets. However, this option was dismissed due to the significant heating of the nebulizer solution and difficulties with nebulizing high-viscosity liquids as the pulmonary surfactant [13]. Vibrating mesh nebulizers force liquid medications through multiple apertures in a mesh to generate aerosol but they still present some challenges. For instance, viscous drugs can clog the pores, cleaning the mesh can be difficult and they are more expensive than the other alternatives, as explained by Pillow et al. [18]. Tiemersma et al. [19] evaluated lung deposition of salbutamol using two vibrating mesh nebulizers, designed specifically for use in preterm infants, and they compared them with a jet nebulizer and a pMDI. The results showed that lung deposition was significantly higher for the investigational vibrating mesh nebulizers in an in vitro model of a preterm infant of 32-weeks gestational age. Recently, Choi et al. [20] manufactured a micro-porous mesh nebulizer with an optimized Pd-Ni membrane filter. Their results showed a good biocompatibility and an excellent durability which makes this nebulizer quite promising for its use in biomedical engineering.

Mazela et al. [21] reported the dilemma with regard to aerosol delivery to preterm infants. This study described the low pulmonary deposition (0.5–1% of the nominal dose) in neonates with either jet nebulizers or pressurized metered-dose inhalers (pMDIs) and how different factors can alter the aerosol delivery in the ventilated infant. Kohler et al. [22] studied the lung deposition in seventeen spontaneously breathing preterm infants with three different nebulizers. They confirmed that, on average, not more than 1% of the nominal dose reached the lungs with the jet and ultrasonic nebulizers employed. Danaei et al. [23] made a review of the impact of particle size on drug delivery in different clinical applications. To date, five clinical studies have been carried out with aerosolized surfactant administration [24,25,26,27,28], showing some discrepancies in the results. The most recent study of Minocchieri et al. [28] suggests minimal adverse effects within the first week of life after nebulization treatment and longer time to reach CPAP failure criteria compared with the control group. Special catheters have been used for the administration of intracorporeal nebulized surfactant. Rey-Santano et al. [29] showed that surfactant delivered as an aerosol, by means of an inhalation catheter, can produce a similar response to rapid intratracheal bolus instillation of the same dose, in terms of gas exchange and pulmonary mechanics, and resulting in less lung damage. Additionally to surfactant, the use of perfluorocarbons (PFCs) has been studied to improve lung function. Murgia et al. [30,31] carried out in vitro studies with three intratracheal inhalation catheters to deliver an aerosol of PFC and surfactant, showing their feasibility for treating the RDS and how ventilation strategies influence in their efficiency. Burkhardt et al. [32] studied the behavior of a PFC-surfactant mixture (Persurf) in depleted rats and observed an improvement in oxygenation as well as a more homogenous distribution compared with the surfactant alone. Goikoetxea et al. [33] reported an enhancement in deposition delivering an intracorporeal aerosol beyond the third generation of neonatal branching by means of an inhalation catheter. Additionally, they studied by a numerical modeling the surfactant aerosol properties within a neonatal physical model and validated the results with experimental data [34]. Syedain et al. [35] developed a novel aerosol generator for surfactant aerosol delivery in preterm infants. Even though this device still requires the intubation of the neonate it showed promising results, with the generation of small aerosol droplets and operating with low airflow. Milesi et al. [36] developed a new atomizing device, consisting of a small multilumen catheter, for intracorporeal nebulization of surfactant during CPAP. In a recent research, they conducted a study to deliver nebulized surfactant without the need of intubation in spontaneously breathing preterm lambs, showing encouraging results [37]. Computational Fluid Dynamics (CFD) tools have also shown great potential to analyze the effects of pharmaceutical aerosols in airway models, as shown in the study of Xi et al. [38]. Aramendia et al. [39] developed a numerical model with CFD techniques to study the particle size and cumulative mass distribution of two different PFC compounds. 

Nebulization can be a useful alternative to tracheal instillation for the treatment of RDS, since it can produce a similar response in terms of gas exchange and pulmonary mechanics [29]. However, discrepancies in the experimental and clinical results have been found [24,25,26,27,28] that can be attributed to several factors, such as the use of different animal models of lung injury or the use of different types of aerosol devices. Therefore, the current research has been performed to study the influence in the particle size distribution of two prototypes with different distal shapes. The aim is to evaluate the feasibility to generate droplets with potential to be delivered in the supraglottic region. We hypothesized that with these nebulizers a PFC aerosol could be generated with particles within the respirable size. To that end, (1) two novel nebulizer prototypes have been designed and manufactured, (2) the particle size distribution with distilled water and two PFCs has been measured with each one, and (3) their performance was analyzed and compared with the results obtained in previous research.

## 2. Materials and Methods

Additive manufacturing techniques, by means of the 3D SYSTEMS-ProJet MJP 5600 printer, were employed for the manufacturing of the prototypes tested in the current study. The Polyjet technology was used, based on the photopolymerization of resin, which consists in the projection of micro droplets over a platform along with an emission of ultraviolet light which solidifies the material with a curing procedure. One of the most indispensable characteristics for the manufacturing of these nebulizers is the generation of the wax support, which is removed with heat after the printing. This technology has a height layer precision of 16 µm, allowing the manufacturing of the holes required in these prototypes between 0.3–0.7 mm. The material used is translucent clear ultraviolet curable plastic with a solid density of 1.18 g/cm^3^.

Few works have studied drug delivery devices for neonates. The design of these prototypes has been carefully carried out after analyzing previous research with different types of inhalation catheters and nebulizers for preterm infant population. For instance, Rey-Santano et al. [29] showed improvements in lung function with aerosol delivery to the same extent than tracheal instillation. Syedain et al. [35] evaluated an intrapulmonary aerosol generation device for surfactant delivery in preterm infants. Holbrook et al. [40] developed several nebulization devices to administer pharmaceutical aerosols to ventilated infants. However, these devices require the intubation of the infant. Goikoetxea et al. [33] studied the performance of an inhalation catheter (Aeroprobe, Trudell Medical International) to deliver surfactant and PFC beyond the third generation of the neonatal airways. Recently, Milesi et al. [36,37] developed a nebulizer to deliver relatively large particles in the subglottic and supraglottic region.

**Figure 1:**
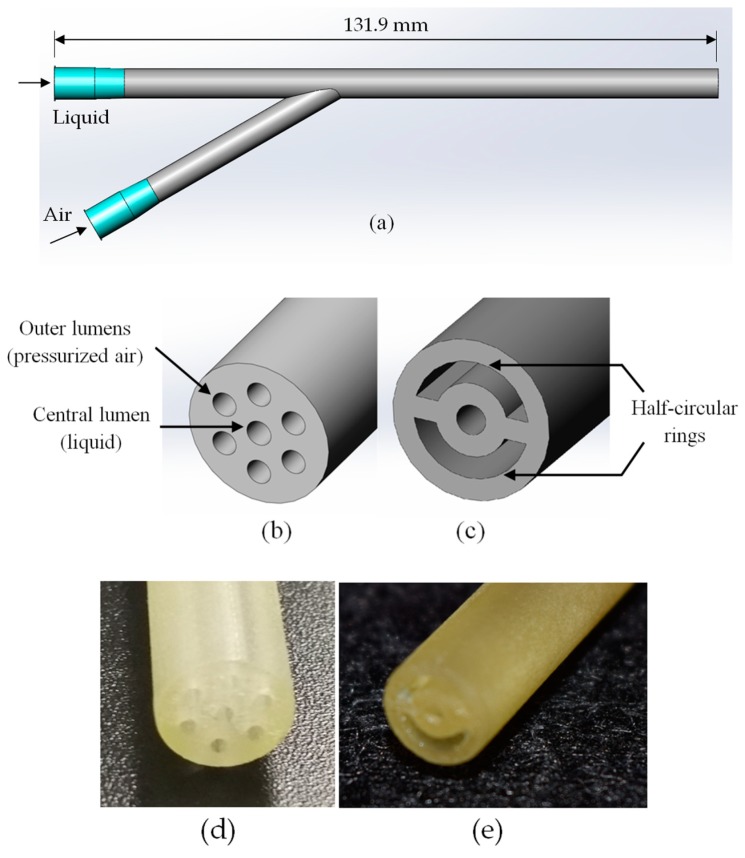
(**a**) 3D visualization of the prototype, distal section of (**b**) prototype 1, (**c**) prototype 2. Distal section of the manufactured (**d**) prototype 1 and (**e**) prototype 2.

For the aforementioned, further research is needed to develop a device intended for neonates in order to optimize the drug delivery in a noninvasive and effective way. Both prototypes designed and presented in the current manuscript are classified within twin-fluid nebulizers with external mixing, that is the gas interacts with the liquid outside of the nebulizer. Twin-fluid nebulizers typically produce a full cylindrical spray cone with relatively small droplets at high liquid mass flow rates [41]. Two different prototypes have been developed that allow aerosol generation, offering potential to drug delivery without the need of intubation. Figure 1a shows a 3D visualization of the prototypes. Prototype 1 consists of six small outer lumens, where compressed air is delivered, and a central lumen where the liquid flows; see Figure 1b. On the other hand, in the prototype 2, the outer lumens have been replaced with two half-circular rings; see Figure 1c. The central lumen has the same dimensions in both prototypes and the area of the two half-circular rings in the prototype 2 is equal to the area of the six outer lumens of the prototype 1. Figure 1d,e show the distal section of the manufactured nebulizers, prototype 1 and prototype 2 respectively. Figure S1 in supplementary information provides a picture of the manufactured nebulizer prototype 2.

Three compounds have been used in order to analyze and compare the operation of the two prototypes presented in the current study, distilled water (H_2_Od) and two different PFCs, perfluorodecalin (PFD; C_10_F_18_, F2 Chemicals Ltd., Lancashire, UK) and FC75 (C_8_F_16_O, Fluorinert, 3M, Neuss, Germany). Their properties can be seen in Table 1. Distilled water is used for drug dilution and the aerosolization of aqueous medications whereas the biophysical properties of PFCs have shown to improve oxygenation and to reduce lung injury in cases of severe respiratory insufficiency, as studied by Guo et al. [42]. Partial liquid ventilation with PFCs has been used in the experimental field and proven effective in the treatment of various lung diseases in a wide range of animal models, reaching human clinical trials [43]. The aerosol delivery of PFCs has been suggested as a promising method over instillation to improve lung function [44]. Von der Hardt et al. [45] compared different PFCs in surfactant-depleted rabbits showing their effectiveness and suitability for aerosol treatment. 

**Figure 2:**
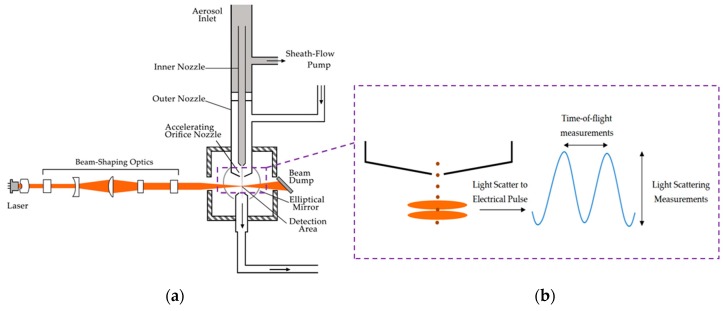
(**a**) Aerodynamic Particle Sizer (APS) operation scheme, (**b**) detail view with aerosol droplets crossing the overlapping beams and generating the double-crested signal.

An Aerodynamic Particle Sizer (APS) spectrometer was used to measure the aerosol produced with both nebulizer prototypes. The aerosol drawn into the inlet is immediately split into a sample flow, through the inner nozzle, and a sheath flow, through the outer nozzle. This device, based on a double-crested optical system for unmatched sizing accuracy, generates a signal every time a particle crosses two laser beams placed within the inlet nozzle, providing high resolution measurements for droplets between 0.5 and 20 µm (see Figure 2). The acceleration of droplets, due to inertia, is smaller for larger droplets. Therefore, the APS theory operation to calculate this acceleration is based on the time between the peaks of the signal produced by the two laser beams, also known as time of flight. Then, the APS memory, which is initially calibrated, converts each time of flight measurement recorded to the corresponding aerodynamic particle diameter, described as the diameter of a spherical particle with a density of a water droplet (1000 kg/m^3^) that has the same settling velocity as the measured particle.

**Figure 3:**
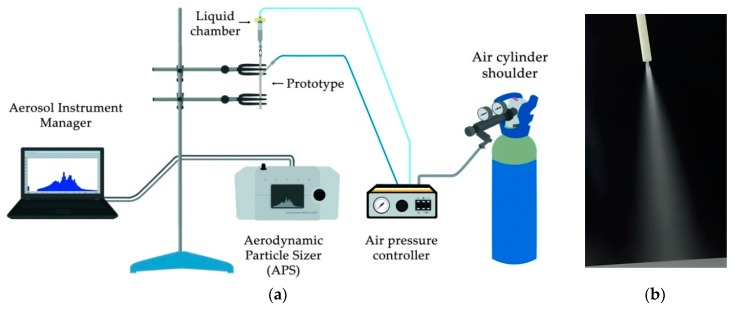
(**a**) Experimental setup used for the particle size characterization and (**b**) an aerosol visualization of a nebulizer prototype.

Figure 3a shows the experimental setup used to measure the particle size distribution of the aerosols generated with both nebulizer prototypes. The mass median aerodynamic diameter (MMAD), the mean aerodynamic diameter (Da) and the geometric standard deviation (GSD) were analyzed. The central inlet of the prototypes, where the liquid is delivered, was connected to a three-way stopcock to control the charge of the compound into the liquid chamber. Then, an air pressure controller was used to provide compressed air to both the small piston placed within the liquid chamber and to the side connection of the nebulizer prototype, where the air is delivered to the outer lumens and to the two half-circular rings respectively. The air was supplied by an air cylinder shoulder with purity higher than 99.999% assuring that the air delivered is empty of impurities that could block the nebulizer prototypes lumens or the APS nozzles. The distance between the inlet nozzle of the APS and the distal section of the nebulizer prototypes was controlled during the experiments in order to get a value as close as possible to the average particle concentration recommended by the device guidelines of 1000 droplets/cm^3^. The measurements recorded by the APS are classified in four categories according to their aerodynamic diameter value. In the first category, those droplets with a diameter smaller than 0.5 µm, in the second one the droplets that are within the spectrometer measuring range from 0.5 µm to 20 µm, in the third category the droplets that cross the laser beams at the same time and cannot be sized, and in the last category those droplets larger than the measuring range of 20 µm. Thus, it was important to check that in every sample taken most of the droplets were classified in the second category, which comprises the droplets within the measuring range of the APS. All the samples were recorded, stored and analyzed by the Aerosol Instrument Manager software associated with the APS. Figure 3b provides an example of the aerosol visualization obtained with prototype 1. Additionally, in Figure S2 of supplementary information an aerosolization example with prototype 2 can be found. Figures S3 and S4 show the experimental setup placed in the laboratory for the nebulization with both prototypes.

### Statistics

A statistical analysis was carried out with the data obtained. The measurements were repeated five times for each compound and prototype and the mean ± standard deviation were calculated for Da, MMAD and GSD. A one-way analysis of variance (ANOVA) was performed to identify any statistically significant differences between the prototypes and each compound for aerosolization rates with a *p* < 0.05 accepted as significant.

## 3. Results

**Figure 4:**
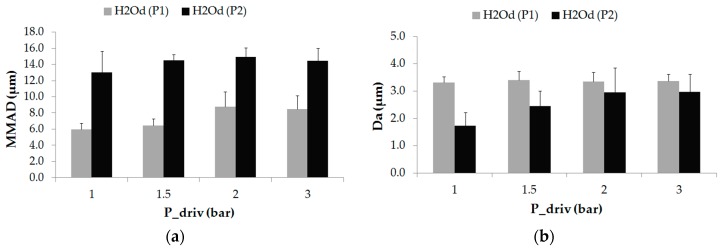
(**a**) MMAD (µm) and (**b**) Da (µm) values for prototype 1 (P1) and prototype 2 (P2) as a function of the driving pressure for distilled water (H_2_Od). Values are given as mean ± standard deviation.

In the present study, the characterization of the aerosol was studied by means of the MMAD, Da and GSD. The MMAD measures the aerodynamic diameter at which 50% of the aerosol mass is present in droplets below this value. The aerosols produced by prototype 1, during distilled water aerosolization, provided MMAD values lower than 9 µm. Specifically, a minimum value was observed with a driving pressure of 1 bar (5.96 ± 0.76 µm) and a maximum value with a driving pressure of 2 bar (8.80 ± 1.84 µm), see Figure 4a. On the contrary, larger MMAD measurements were achieved during aerosolization with prototype 2 with values up to 15 µm. A minimum value with a driving pressure of 1 bar (13.03 ± 2.62 µm) and a maximum peak with a driving pressure of 2 bar (14.93 ± 1.13 µm) were obtained. It should be noted that for both prototypes the minimum and maximum MMAD values were obtained for the same driving pressures, 1 and 2 bar respectively. 

The aerodynamic diameter is described as the diameter of a spherical particle with a density of a water droplet (1000 kg/m^3^) that has the same settling velocity as the particle. The results obtained with prototype 1 and distilled water maintained a stable value regardless of the driving pressure, between 3.32 ± 0.20 µm (1 bar) and 3.40 ± 0.31 µm (1.5 bar). Prototype 2, however, presented a smooth increment in the aerodynamic diameter proportionally with the driving pressure, as shown in Figure 4b.

The GSD is a dimensionless number which provides an indication of the spread of sizes of droplets that form the aerosol. An aerosol with a GSD value below 1.2 indicates that the aerosol is formed by droplets with the same or very nearly size (monodisperse aerosol), whereas a value above 1.2 shows that the aerosol contains droplets of many different sizes (heterodisperse aerosol), as defined by O’Callaghan et al. [16]. Both prototypes produced heterodisperse aerosols, as showed in Table 2, with GSD values between 1.79 ± 0.03 (prototype 1 at 1 bar) and 2.47 ± 0.28 (prototype 2 at 2 bar).

**Figure 5:**
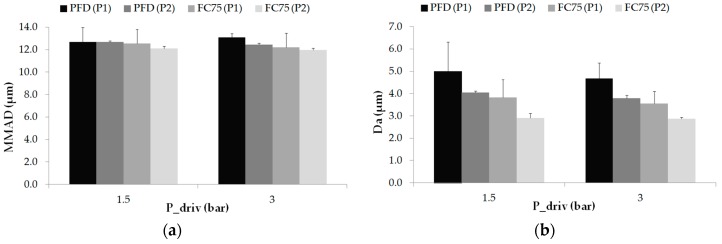
(**a**) MMAD (µm) and (**b**) Da (µm) values for prototype 1 (P1) and prototype 2 (P2) as a function of the driving pressure for PFD and FC75. Values are given as mean ± standard deviation.

After the preliminary tests carried out with distilled water, PFCs were tested in order to study more in detail the behavior of both prototypes under a compound that can be used subsequently as a mixture with surfactant. With regard to the MMAD values, the results with both prototypes showed relatively uniform values, close to 12 µm, for the two driving pressures considered. The largest value (13.08 ± 0.36 µm) was obtained with PFD and prototype 1 applying a driving pressure of 3 bar whereas prototype 2 with FC75 provided a minimum value of 11.96 ± 0.17 µm (see Figure 5a). The large difference in the MMAD results obtained, compared with those under distilled water, may be caused due to the high volatility of the PFCs compounds. The Da in all the cases tested was below 5 µm, as represented in Figure 5b. The results with prototype 2 were slightly lower than those with prototype 1 for each case and driving pressure. In addition, larger values were achieved with PFD irrespective of the prototype geometry and the pressure defined. The two half-circular rings surrounding the prototype 2, where the air is delivered, instead of the circular lumens of prototype 1 may contribute to a larger breakup of the PFCs and, therefore, the generation of smaller droplets. The largest value was achieved with prototype 1 and PFD (5.00 ± 1.38 µm) whereas prototype 2 with FC75 provided the minimum value (2.87 ± 0.07 µm). In addition, for the same pressure and the same prototype, the size of the droplets is smaller for the case with FC75 in comparison with PFD, as shown in Figure 5b. The reason could be found in the fact that the vapor pressure of the FC75 (63 mmHg) is four times larger than the PFD (14 mmHg). In the case of FC75, the nebulization might lead to the development of smaller droplets or even the generation of PFC vapor [31,45].

As happened with distilled water, both prototypes produced heterodisperse PFC aerosols with GSD values between 2.00 ± 0.08 µm (prototype 2 with FC75) and 2.34 ± 0.02 (prototype 2 with PFD), as detailed in Table 3.

## 4. Discussion

The potential advantages of minimally invasive techniques in the treatment of different respiratory diseases has emerged the study of alternative methods as the aerosolization. The current experimental study was carried out to evaluate and compare two novel nebulizer prototypes to generate respirable droplets with potential to be delivered in the supraglottic region. The prototypes were created by additive manufacturing techniques and subsequently tested with an APS in order to measure the particle size distribution.

This study reflects the importance of the nebulizers’ geometry and how it can affect in the aerosol size distribution. A main finding of the evaluation of these two novel nebulizer prototypes was that they produced droplets with aerodynamic diameters within the optimal range recommended of Da = 1–5 µm for its correct inhalation [46]. In the upper respiratory tract, droplets of 2–5 µm are desirable. Particles of size larger than 5 µm are mainly deposited by impaction in the oropharyngeal region and are unable to reach to the lungs, whereas particles of size smaller than 1 µm are mostly exhaled without deposition. It is also important to note that the MMAD values obtained were between 6–13 µm and, therefore, a high amount of the aerosol is transported by droplets larger than 5 µm. Nevertheless, a larger MMAD values might not be a problem taking into account that the aim of these prototypes is to be placed in the supraglottic region. Due to the spreading properties of viscous substances as the surfactant, droplets with larger diameters than 5 µm could reach the lungs along with finer particles that could penetrate further into the smaller airways for deep lung delivery [36]. With regard to prototype 1, our findings with distilled water showed similar values in terms of Da compared with those obtained by Goikoetxea et al. [33] using the Aeroprobe pneumatic catheter. In addition, PFCs provided slightly larger Da values with both prototypes. In this study, the Da value varies in a range of 3–5 µm while in Goikoetxea et al. [33] values approximately of 2 µm were measured. This increment could be benefit to obtain a better deep lung deposition. The difference in MMAD with PFCs between both studies is more pronounced, since the prototypes presented in the current study provided most of the aerosol mass distributed in droplets within 10–20 µm. The results with the Aeroprobe pneumatic catheter showed lower MMAD values even though the experiments were also carried out at higher driving pressures (4–7 bar). On the other hand, an important difference in particle size distribution was observed with the atomizing system of Milesi et al. [37], where droplets with median diameter of 40–60 µm were measured at the tip of their catheter tip. 

**Figure A1:**
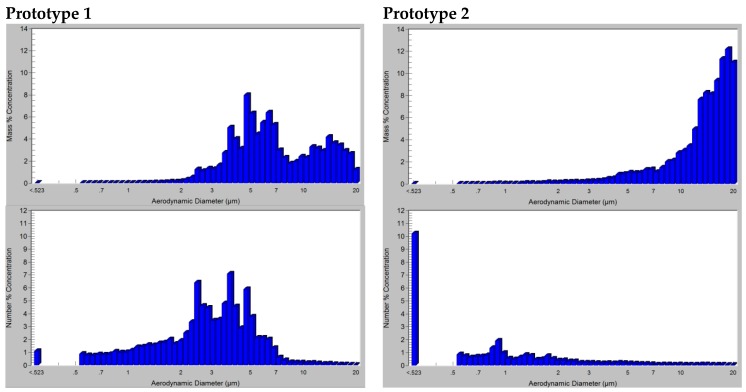
Mass and number distribution of an H_2_Od aerosol sample with prototype 1 (**left**) and prototype 2 (**right**). (Screenshots taken directly from the Aerosol Instrument Manager software).

Figure A1 of the Appendix A represents the particle mass and number distribution of one of the aerosol samples taken for distilled water with each prototype and the same driving pressure. From there, it can be visualized how the particle mass concentration with prototype 1 is transported by droplets in a wider range than prototype 2, where most of the aerosol mass is formed by droplets of 10–20 µm. However, taking into account the concentration number, prototype 2 presents most of the droplets classified below 3 µm, which is in concordance with the difference between the values of Da and MMAD explained in the results. The different behavior of prototype 2 with respect to prototype 1 may be attributed to the half-circular ring geometry proposed for the air delivery and its possible influence in the breakup and coalescence of the aerosol droplets.

The study presents some limitations that must be acknowledged. During the particle size distribution measurements it was observed that the aerosolization rate was too high to nebulize the 5 mL of compound contained in the liquid chamber in each sample. This issue is directly related with the dimensions of the nebulizer lumens of the prototypes manufactured, which were adjusted as close as possible to the limitations of the 3D printer. The dimensions of the lumens in the distal tip of the prototypes are being currently adjusted for subsequent studies in order to produce an aerosol with lower aerosolization rates, i.e., an increase in the aerosolization time to administer a dose. This will also lead to a reduction in the distal pressure, which is indispensable not to exceed a value of 10 cm H_2_O suggested by neonatologists. All these aspects should be addressed in the development of new improved prototypes and in future experimental studies.

## 5. Conclusions

The constant investigation of new minimally invasive techniques to treat the respiratory distress syndrome has lead to the research of alternative approaches as the aerosolization. Since clinical studies to date have shown discrepancies in the results, different research lines are under study to evaluate new aerosol devices, their placement within the respiratory system and variations in the compound preparations and doses. In the present work, the aerosol generation of two novel nebulizer prototypes, with different distal geometries, has been evaluated with distilled water and two PFCs.

Overall, the current study demonstrates the feasibility of these novel jet nebulizers to produce an aerosol and generate droplets of respirable size. However, differences have been observed between the two different geometries proposed. Prototype 1 produces a wider mass distribution than prototype 2, which provides most of the particle mass distribution in droplets between 10–20 µm, even though there is an increment in the number of smaller droplets with respect to prototype 1. Consequently, distal shapes with outer lumens similar to prototype 1 seem to be more suitable for the generation and delivery of a uniform aerosol than prototype 2. 

## Figures and Tables

**Table 1:** Properties of H_2_Od and perfluorocarbons (PFCs).

Parameter	H_2_Od	PFD	FC75
Density (ρ) [g/mL]	0.99	1.95	1.78
Kinematic viscosity (ν) [cSt]	1.003	2.70	0.81
Surface tension (γ) [dyn/cm]	73	15	15

**Table 2:** Geometric standard deviation (GSD) for both prototypes nebulizing H_2_Od at different pressures.

P (bar)	PROTOTYPE 1 ^(a)^	PROTOTYPE 2 ^(a)^
**1**	1.79 ± 0.03	1.95 ± 0.31
**1.5**	1.86 ± 0.05	2.31 ± 0.26
**2**	1.86 ± 0.06	2.47 ± 0.28
**3**	1.90 ± 0.05	2.37 ± 0.12

^(a)^ Values are given as mean ± standard deviation.

**Table 3:** Geometric standard deviation (GSD) for both prototypes nebulizing PFD and FC75 at different pressures.

	PROTOTYPE 1 ^(a)^		PROTOTYPE 2 ^(a)^	
P (bar)	PFD	FC75	PFD	FC75
**1.5**	2.23 ± 0.14	2.26 ± 0.12	2.34 ± 0.02	2.00 ± 0.08
**3**	2.24 ± 0.10	2.25 ± 0.08	2.29 ± 0.02	2.04 ± 0.02

^(a)^ Values are given as mean ± standard deviation.

## Supplementary Materials

The following are available online at http://www.mdpi.com/1999-4923/11/1/19/s1, Figure S1: Representation of the nebulizer prototype 2; Figure S2: Nebulization example with prototype 2; Figure S3: Experimental setup placed in the laboratory for the nebulization with both prototypes; Figure S4: (a) Setup of the nebulizer with the liquid chamber where the liquid is supplied and (b) detail of the connections to administer the compressed air and the liquid respectively.
