# Supplementary material for: Experimental Evaluation of Perfluorocarbon Aerosol Generation with Two Novel Nebulizer Prototypes
Source: Pharmaceutics. 2019 Jan 5;11(1):19. doi: 10.3390/pharmaceutics11010019 (PMC6358822; doi:10.3390/pharmaceutics11010019)
Supplement: Supplementary file 1 [file pharmaceutics-11-00019-s001.pdf]

pharmaceutics

MDPI

Supplementary Materials: Experimental Evaluation of Perfluorocarbon Aerosol Generation with Two Novel Nebulizer Prototypes

Iñigo Aramendia, Unai Fernandez-Gamiz, Alberto Lopez-Arraiza, Carmen Rey-Santano, Victoria Mielgo, Francisco Jose Basterretxea, Javier Sancho and Miguel Angel Gomez-Solaetxe

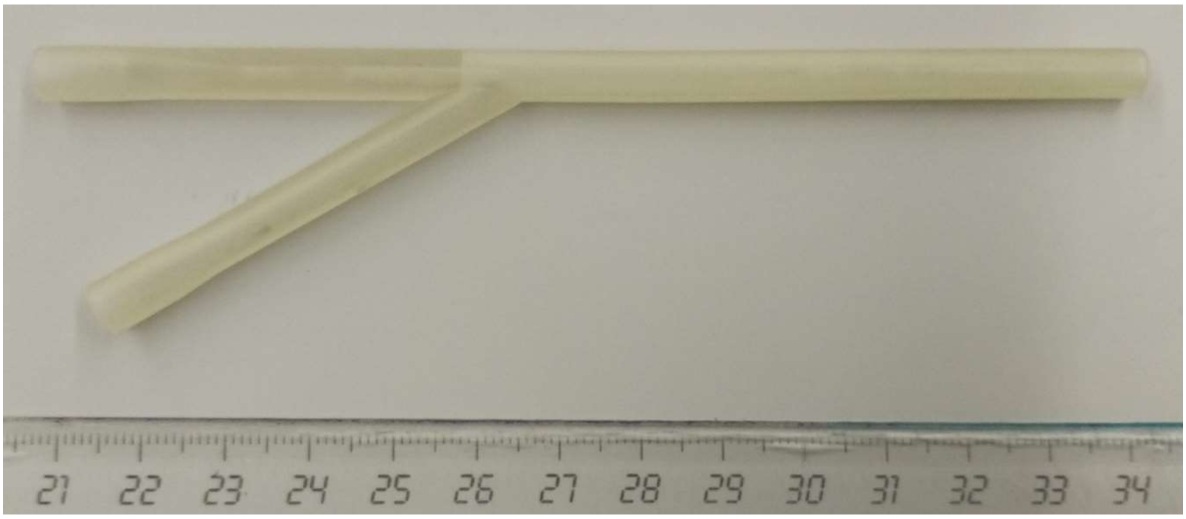

Figure S1. Representation of the nebulizer prototype 2.

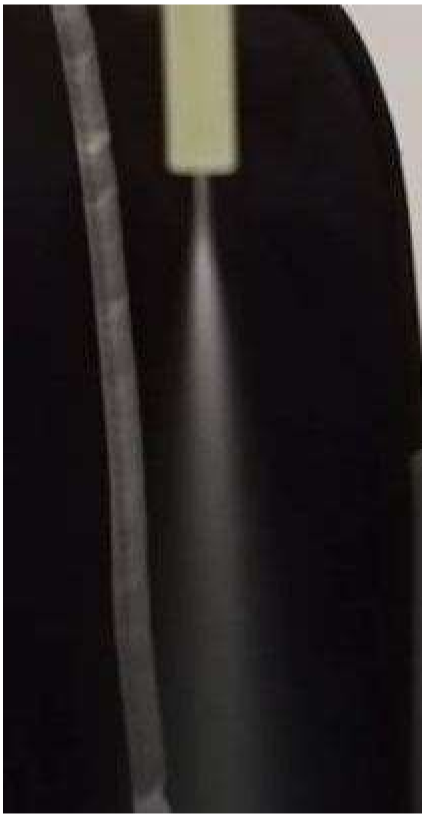

Figure S2. Nebulization example with prototype 2.

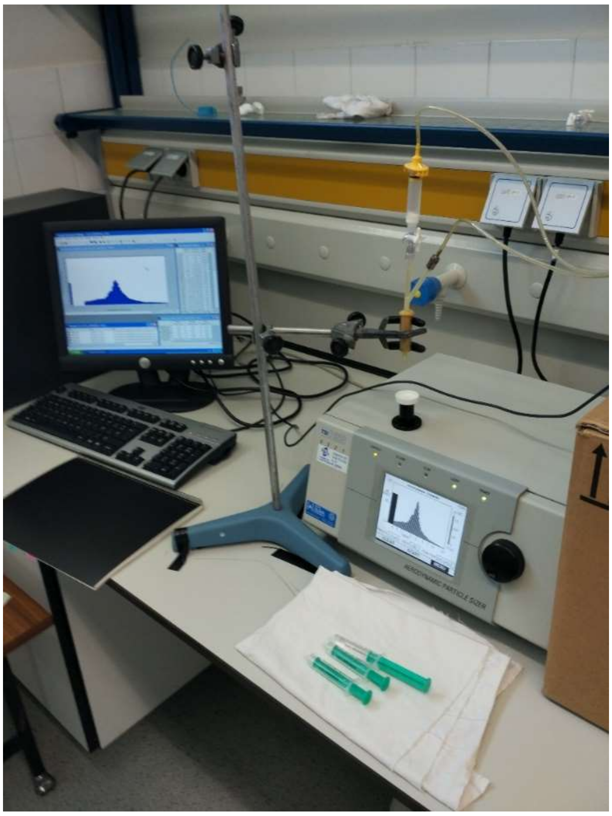

Figure S3. Experimental setup placed in the laboratory for the nebulization with both prototypes.

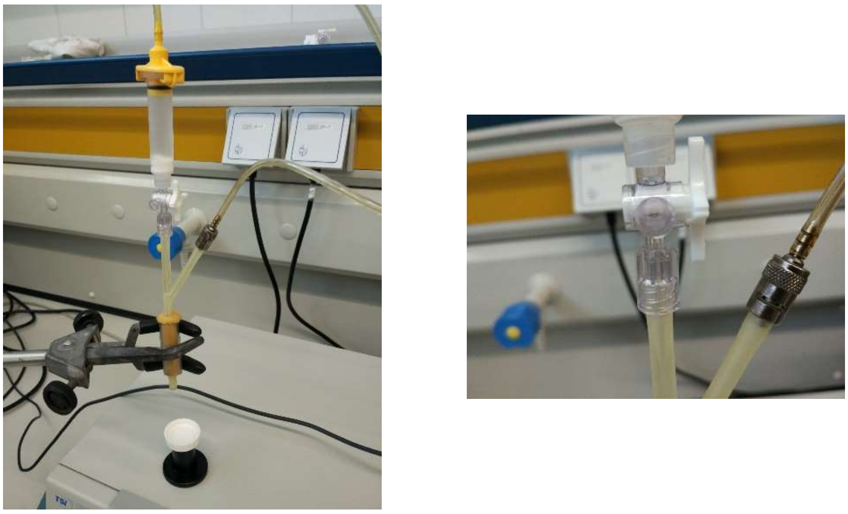

(**a**) (**b**)

Figure S4. (**a**) Setup ofthe nebulizer with the liquid chamber where the liquid is supplied and (**b**) detail of the connections to administer the compressed air and the liquid respectively.
